# Improved Electromagnetic Interference Shielding Efficiency of PVDF/rGO/AgNW Composites via Low-Pressure Compression Molding and AgNW-Backfilling Strategy

**DOI:** 10.3390/nano14181531

**Published:** 2024-09-21

**Authors:** Zhunzhun Li, Yaqun Li, Zhusong Mao, Xingyu Mei, Qimei Zhang

**Affiliations:** School of Materials and Environmental Engineering, Chizhou University, Chizhou 247000, China; lizz@czu.edu.cn (Z.L.); l039767@outlook.com (Y.L.); mrs2915407138@outlook.com (Z.M.); a15883000871@outlook.com (X.M.)

**Keywords:** electromagnetic interference shielding, silver nanowires, polyvinylidene fluoride, polymer composites

## Abstract

Silver nanowires (AgNWs) have excellent electrical conductivity and nano-sized effects and have been widely used as a high-performance electromagnetic shielding material. However, silver nanowires have poor mechanical properties and are prone to fracture during the preparation of composite materials. In this study, PVDF/rGO/AgNW composites with a segregated structure were prepared using low-pressure compression molding and the AgNW-backfilling process. The low-pressure compression of the composite significantly improves its electromagnetic shielding performance because the low-pressure process can maintain the AgNWs’ integrity. The backfilled AgNWs played a vital role in increasing the path of electromagnetic wave propagation and the absorption of electromagnetic waves. The backfilled amount of AgNWs was only 1 wt%, which increased the composite material’s conductivity by one order of magnitude. The total electromagnetic interference shielding (SE_T_) of the composite materials increased by 23.3% from 24.88 dB to 30.67 dB. The absorption contribution (SE_A_/SE_T_) increased from 84.2% to 92.8%, significantly improving the electromagnetic interference shielding and the absorption contribution of the AgNWs in the composites. This was attributed to the backfilling of the porous structure by the AgNWs, which promoted multiple reflections and enhanced the absorption contribution.

## 1. Introduction

Electromagnetic waves, which are essential for modern communications, offer both benefits and challenges. Electromagnetic interference (EMI) pollution can harm precision equipment, which is a problem [1,2]. EMI contamination can potentially damage biological cells and genes via thermal and non-thermal effects and increase the risk of cancer by affecting the body’s immune system and metabolism [3]. High-performance electromagnetic shielding materials, especially absorption-dominated ones, are crucial in reducing EMI contamination by maintaining the stable operation of electronic devices and protecting the health and safety of humans and organisms.

In response to practical needs, metallic materials with poor stability and secondary reflection have been phased out, and lightweight, corrosion-resistant, and stable EMI shielding conductive polymer composites (CPCs) have been adopted instead [4,5]. Numerous high-quality conductive fillers, such as carbon nanotubes (CNTs) [6], graphene [7], MXenes [8,9], metal nanoparticles, and metal nanowires, have been extensively utilized to enhance the EMI shielding effectiveness (EMI SE) of polymer composites.

Metal nanowires are particularly effective in improving the EMI SE of CPCs. Despite their high cost, AgNWs are still widely used in the electromagnetic shielding field [10,11,12]. For example, AgNWs and graphene are used as functional fillers to prepare composite films with a high electromagnetic shielding performance [3]. Recently, researchers have shifted their focus from simply enhancing the EMI of silver/polymer composites by controlling the reflection of microstructures to designing multifunctional electromagnetic interference shielding materials based on silver/polymer composites, focusing on improving absorption loss while minimizing reflection loss [13]. The current research on conductive composite materials believes that a segregated structure is an effective approach to reducing the amount of conductive filler needed [14].

When combined with a polymer, AgNWs overlap with each other to form an efficient conductive network due to their high aspect ratio, thus giving the composite excellent electrical conductivity [15]. However, the AgNWs are subjected to stress during the composite’s processing, which may cause fracture [16,17]. This reduces its aspect ratio and adversely affects the composite’s conductivity, thereby reducing its electromagnetic shielding effectiveness. The low-pressure molding method reduces the damage to AgNWs during their processing.

In this study, we utilized the low-pressure compression molding method to fabricate PVDF/rGO/AgNW composites with a segregated structure, aiming to minimize the damage to the AgNWs. On this basis, AgNWs were infused into the composites via a vacuum-backfilling process to enhance the conductive network of the AgNWs within the segregated structure. This innovative approach has practical applications. The low-pressure technique decreases the breakage of AgNWs, preserving the network integrity and improving conductance. The strategic incorporation of backfilling solidifies the segregated structure, offering additional support and stability to the AgNW network, and further optimizes the composite structure, resulting in a backfilling porous structure. This enhanced conductivity and connectivity improves the electromagnetic shielding, effectively increasing the absorption capabilities. The improved shielding highlights the potential to reduce electromagnetic wave pollution in various applications.

## 2. Materials and Methods

### 2.1. Materials

Polyvinylidene fluoride (PVDF, FL2000) was provided by Zhejiang Fluorine New Chemical Materials Co., Ltd., Shaoxing, China. Polyvinyl pyrrolidone (PVP, K90) and 9, 10-Dihydro-9-Oxa-10-Phosphaphenanthrene-10-Oxide (DOPO, 98%) were purchased from Shanghai Macklin Biochemical Technology Co., Ltd., Shanghai, China. Anhydrous ethanol (AE, 99.7%), Ethylene glycol (EG, 99.5%), vitamin C (99.7%), Sodium chloride (NaCl, 99.5%), and Silver nitrate (AgNO_3_, 99.7%) were purchased from Sinopharm Chemical Reagent Co., Ltd., Shanghai, China.

### 2.2. Preparation

#### 2.2.1. Preparation of AgNWs

AgNWs were prepared using a simple but effective polyol thermal reaction method, similar to Jiang’s method [18]. The specific operation process was as follows: Initially, 25 mL of EG was poured into a 100 mL beaker, followed by the addition and dissolution of 0.73 g of PVP, 2.5 mg of NaCl, and 0.2 g of AgNO_3_ into the beaker. Subsequently, the solution was transferred to a hydrothermal reactor, placed in an oven at 150 °C for 8 h, and then cooled down. Next, the product was mixed with 20 mL of acetone and stirred to obtain sediment, followed by centrifugation and washing with water (at 8000 r/min for 20 min) three times and repeated twice with absolute ethanol. Finally, the AgNWs were dispersed in anhydrous ethanol, and the concentration was calibrated.

#### 2.2.2. Preparation of Graphene Oxide (GO)

Graphene oxide (GO) was synthesized using the modified Hummers method [19]. An explicit description of the synthesis has been presented in previous papers [20].

#### 2.2.3. Preparation of PVDF@rGO@AgNWs Composite Powder

First, PVDF powder (2 g) was dispersed in absolute ethanol, followed by the addition of a graphene oxide (GO) suspension (21.1 mL; 8.96 mg/mL) and a vitamin C (VC) solution (6 mL; 100 mg/mL). The mixture was stirred vigorously at 80 °C for 4 h. The GO was restored to rGO via VC. Then, the resulting product was thoroughly washed and filtered to obtain a PVDF@rGO composite powder. Subsequently, the PVDF@rGO composite powder was dispersed in a AgNW/ethanol dispersion (16.9 mL; 5.2 mg/mL) under stirring at room temperature for 20 min. Finally, the mixture was centrifuged and dried to obtain a PVDF@rGO@AgNW composite powder consisting of 5 wt% rGO and 4 wt% AgNWs, denoted as PVDF/5rGO/4AgNW composite powder. The rGO content (wt%) was calculated from the mass of GO and the thermogravimetric (TGA) tests of GO, as in a previous work [20].

#### 2.2.4. Preparation of PVDF/rGO/AgNWs Composites

##### PVDF/rGO/AgNW Composite Prepared via High-Pressure Molding

The PVDF@5rGO@4AgNW composite powder was added into a mold, preheated at 170 °C for 20 min, and then molded under 12 MPa at 170 °C for 20 min. The obtained composite with a thickness of 2.2 ± 0.3 mm was denoted as PVDF/rGO/AgNWs−H.

##### PVDF/rGO/AgNW Composite Prepared Using Low-Pressure Molding

The PVDF@5rGO@4AgNW composite powder was added into a mold and preheated at 175 °C for 20 min and then molded under 7 MPa at 175 °C for 20 min. The obtained product with a thickness of 2.2 ± 0.3 mm was denoted as PVDF/rGO/AgNWs−L.

##### PVDF/rGO/AgNW Composite Prepared Using Backfilling Process

PVDF/rGO/AgNW−L was immersed in a AgNW/ethanol dispersion (10 mL; 5.2 mg/mL) and then transferred to a vacuum-drying oven at 100 Pa at room temperature for 2 h. The samples were washed with anhydrous ethanol and then dried at 70 °C for 4 h. The obtained product was denoted as PVDF/rGO/AgNWs−B. The filling of the silver nanowire was 1wt% and was calculated based on a comparison of the sample masses before and after the filling process.

**Figure 1:**
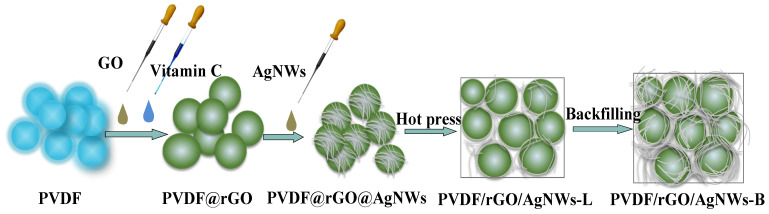
A schematic diagram of preparing PVDF/rGO/AgNWs−B.

PVDF/rGO/AgNWs−L and the PVDF/rGO/AgNWs−B composite are schematically illustrated in Figure 1.

### 2.3. Characterization

A scanning electron microscope (SEM, Phenom Pro, Thermo Fisher Scientific, Bleiswijk, The Netherlands) was used to observe the morphologies of the composite powder and composites. An X-ray diffractometer (XRD, DX-2700, Dandong Haoyuan Instrument Co., Ltd., Dandong, China) was utilized to examine and scan the crystalline phases of the dry sample with a Cu Kα target under radiation featuring a wavelength of 0.154 nm. This instrument scanned over a diffraction angle (2θ) range of 5° to 90° at a rate of 2° per minute. The conductivity measurements were conducted using a four-probe detector (RTS-4, Guangzhou Four-Probe Technology Co., Ltd., Guangzhou, China). The waveguide method was employed for the EMI shielding test across a frequency spectrum of 8.2 to 12.4 GHz within the X-band, with the samples maintaining dimensions of 10.12 mm × 22.86 mm × 2.2 mm. This test was conducted at 25 ± 5 °C, utilizing a vector network analyzer (ZNB 20, Robert & Schwartz Technologies, Inc., Esslingen am Neckar, Germany).

## 3. Results

### 3.1. Characterization Analysis of Raw Materials and Composite Materials

#### 3.1.1. Characterization of rGO

**Figure 2:**
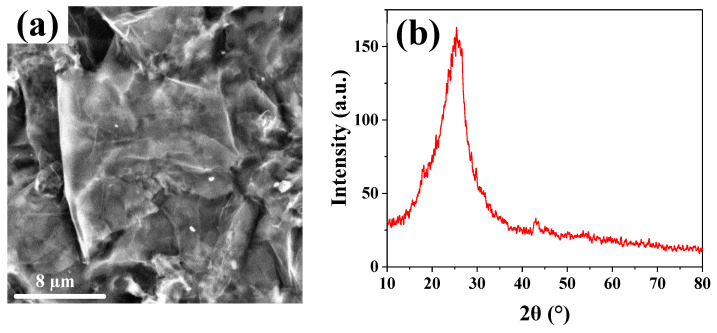
SEM image (**a**) and XRD pattern (**b**) of rGO.

The morphologies of rGO were investigated using SEM. The observational result is shown in Figure 2a. The sample presents a semi-transparent, wrinkled, and curled sheet-like structure, indicating that we successfully prepared few-layer rGO [21]. These layered structures in the SEM image are also relatively large, which is beneficial for the encapsulation of PVDF microspheres. Figure 2b illustrates the XRD spectrum of rGO. A broad peak at 25.4 degrees is observed, corresponding to the (002) crystal plane of graphene [22]. This finding is consistent with the previous literature reports [23]. This morphology also indicates that the studied rGO sample had fine crystal structure characteristics, and the layered structure was relatively intact and had not undergone severe agglomeration or damage [24]. In the analysis of this peak’s formation, the broad peak suggests the possible presence of slight lattice distortions or azimuthal differences between the rGO crystals.

#### 3.1.2. Characterization of AgNWs

**Figure 3:**
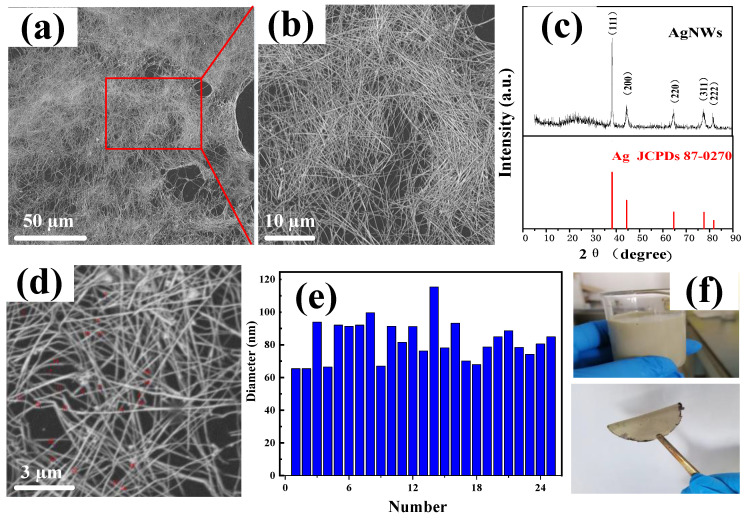
(**a**) SEM images and enlarged view (**b**) of AgNWs; (**c**) XRD patterns of AgNWs; (**d**,**e**) statistical chart of the diameter of AgNWs; (**f**) optical photos of obtained products of AgNWs (**top**) and filter membrane of AgNWs (**bottom**).

Figure 3a,b are SEM images and enlarged views of the AgNWs. The AgNWs had a regular morphology, a uniform size, a length exceeding 60 μm, an average diameter of 82.73 nm (Figure 3d,e), and an aspect ratio of approximately 700. This facilitated the interconnection between the AgNWs to form a conductive network. Figure 3c shows the X-ray diffraction pattern of the AgNWs. The diffraction peaks observed at the 2θ position of 38.32°, 44.62°, 64.64°, 77.58°, and 81.66° correspond to the face-centered cubic phase of the metallic silver (111), (200), (220), (311), and (222) planes [25], respectively, which agrees with the JCPDS card number 87-0720. The high diffraction intensity of the (111) crystal plane suggests that the AgNWs had oriented growth along the (111) crystal plane, indicating the prepared sample was a single-phase nanosilver with a cubic structure. The relative intensity of the diffraction peak on the (111) plane is much higher than that of other peaks, suggesting that the growth rate of the Ag crystals along the (111) plane may have been faster than on the other planes, resulting in a nanosilver with a linear structure [26]. Figure 3f shows the optical photos of the AgNW/ethanol dispersion (top) and the filter membrane of AgNWs (bottom). The AgNWs were relatively stable in the ethanol dispersion, and there was no significant agglomeration after filtration.

#### 3.1.3. The Morphologies of PVDF/5rGO/4AgNW Composite Powder and PVDF/rGO/AgNW Composites

**Figure 4:**
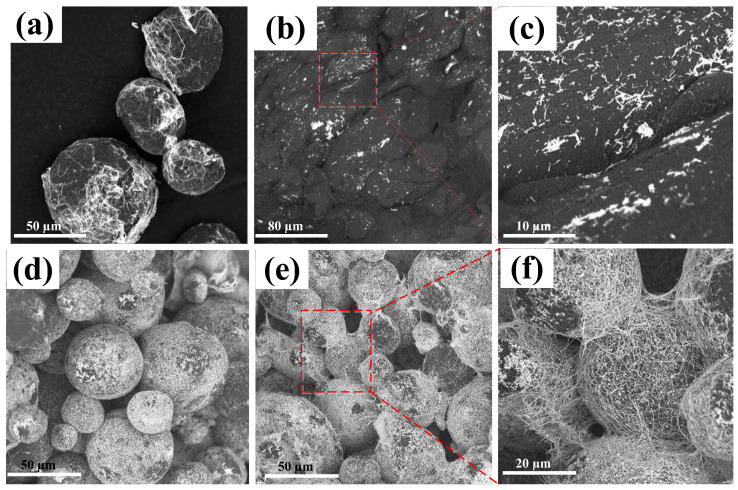
SEM images of PVDF/5rGO/4AgNW composite powder (**a**), PVDF/rGO/AgNWs−H (**b**,**c**), PVDF/rGO/AgNWs−L (**d**), and PVDF/rGO/AgNWs−B (**e**,**f**).

Figure 4 depicts the SEM images of the PVDF/5rGO/4AgNW composite powder and the composite materials prepared with it. Figure 4a shows that the AgNWs and rGO were uniformly coated on the surface of the PVDF microspheres. The length of the AgNWs was approximately 60 μm. The morphology of PVDF/rGO/AgNWs−H is examined in Figure 4b,c. The AgNWs’ microstructure on the PVDF surface significantly changed after the high-pressure shaping process. The reduction in the length of the AgNWs from approximately 60 μm before molding to less than 10 μm was attributed to the high pressure during the molding process [16,17]. As a result, it was difficult to establish an effective conductive network of AgNWs. Simultaneously, the PVDF microspheres were extruded and fused to form a dense PVDF sheet during the high-pressure molding process, making it impossible to use a vacuum-backfilling process to inject the AgNWs.

The SEM image of PVDF/rGO/AgNWs−L is depicted in Figure 4d. Several micropores were formed between partially separated conductive microspheres after low-pressure molding, and PVDF/rGO/AgNWs−L had not yet established a complete conductive network. The composite microspheres showed no significant deformation, with maximum, minimum, and average spacings of 71.7 μm, 7.52 μm, and 28.2 μm between the composite microspheres, respectively. The SEM images indicate that low-pressure molding is beneficial for the size stability of silver nanowires.

The SEM images of the PVDF/rGO/AgNWs−B are shown in Figure 4e,f. After backfilling with AgNWs, the conductive microspheres of PVDF/rGO/AgNWs−B were connected with injected AgNWs to form a fully optimized conductive network, which enables excellent conductivity. Moreover, the filling of AgNWs in the composite materials overlapped with each other at the micropores between the microspheres to form a porous structure.

### 3.2. Composites’ Electrical Conductivity

**Figure 5:**
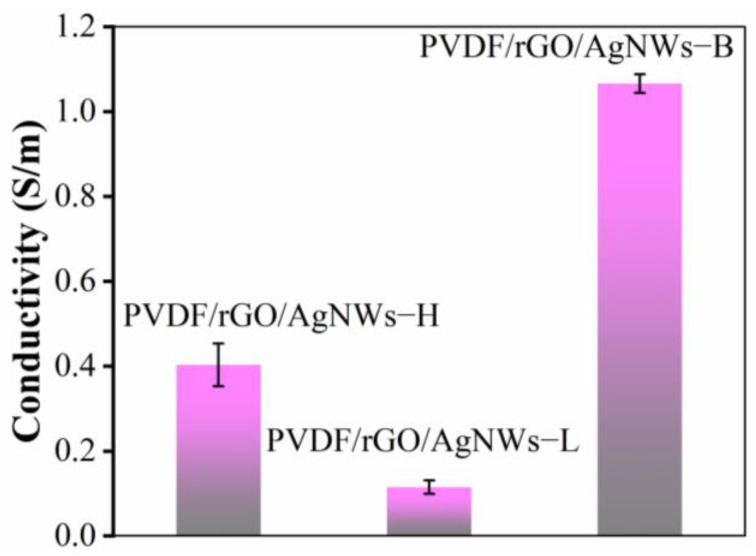
Conductivity of composites based on different processes.

Figure 5 displays the conductivity values of the composites produced using various fabrication techniques. Figure 5 depicts the damage to the silver nanowires under high pressure, resulting in a conductivity value of 0.40 S/m for PVDF/rGO/AgNWs−H. Although silver nanowires experienced less damage under lower pressure, the microporous structure within the composite material disrupted the conductive network, yielding a conductivity of 0.12 S/m for PVDF/rGO/AgNWs−L, which was not higher than that of PVDF/rGO/AgNWs−H. Incorporating AgNWs via backfilling treatment enhanced the interconnectivity of the individually dispersed conductive microspheres, establishing a comprehensive conductive network and, subsequently, augmenting the composite material’s conductivity. As a result, the conductivity of PVDF/rGO/AgNWs−B was measured at 1.07 S/m.

### 3.3. EMI Shielding and Absorption Contribution

According to Schelkunoff’s theory of electromagnetic wave interface conduction, when electromagnetic waves interact at the surface of the prepared nanocomposite, the shielding effectiveness (SE) of the electromagnetic shielding materials consists of three components: surface reflection loss (SE_R_), internal absorption loss (SE_A_), and multiple internal reflection attenuation (SE_M_) for the remainder. The overall electromagnetic shielding effectiveness SE_T_ is the sum of SE_R_, SE_A_, and SE_M_ [27,28].

**Figure 6:**
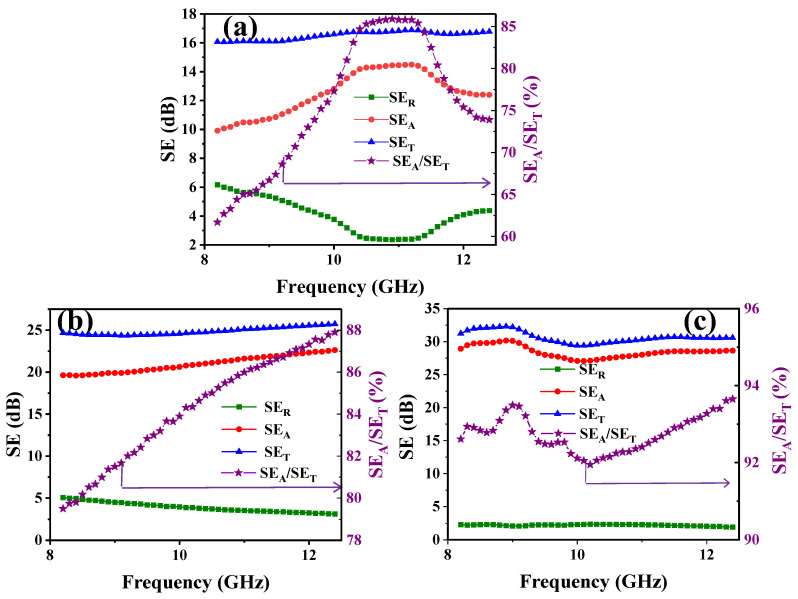
The electromagnetic shielding effectiveness (SE) of PVDF/rGO/AgNWs−H. (**a**), PVDF/rGO/AgNWs−L (**b**), and PVDF/rGO/AgNWs−B (**c**).

The electromagnetic shielding properties of the composites were investigated in the X-band (8.2–12.4 GHz). Figure 6a shows the electromagnetic shielding performance of PVDF/rGO/AgNWs−H. It demonstrated an SE_R_ of 3.99 dB, SE_A_ of 12.5 dB, and SE_T_ of 16.5 dB. The absorption contribution of the composite was 75.70%. In Figure 6b, PVDF/rGO/AgNWs−L shows an SE_R_ of 3.91 dB, SE_A_ of 21.0 dB, and SE_T_ of 24.9 dB. Compared with the data of PVDF/rGO/AgNWs−H, the SE_R_ slightly decreased, while the SE_A_ and SE_T_ increased by 68.0% and 50.9%, respectively. The absorption contribution rose to 84.2%, reflecting an increase of 11.8%. These results suggest that the internal porousness of low-pressure-molded composites can significantly enhance the absorption contribution via multiple loss mechanisms. As shown in Figure 6c, the SE_R_, SE_A_, and SE_T_ of PVDF/rGO/AgNWs−B became 2.22 dB, 28.5 dB, and 30.7 dB after the backfilling process, respectively, with reduced reflection loss, increased absorption loss, and an absorption contribution of up to 92.8%. This may be because the added AgNWs refined the AgNW network by filling the interstices and imperfections, thereby intensifying the conductivity network density and further enhancing the conductivity [29].

### 3.4. Electromagnetic Parameter Analysis

**Figure 7:**
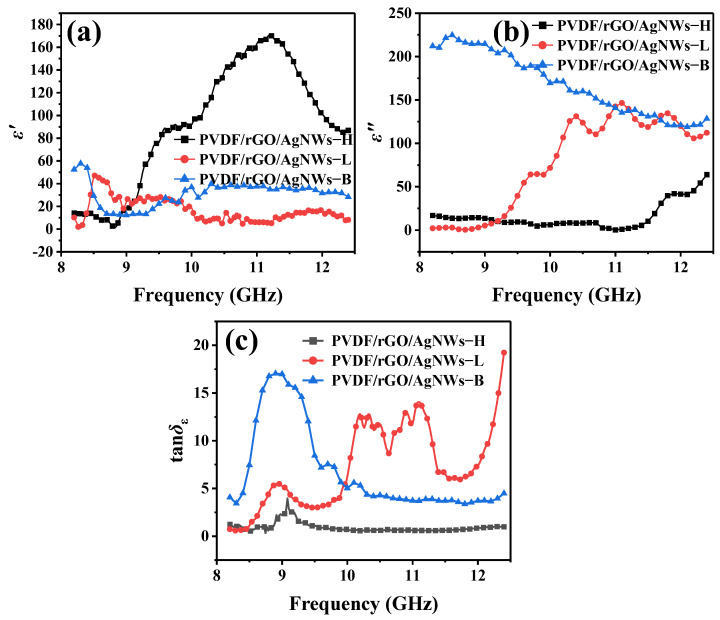
The (**a**) real and (**b**) imaginary parts and (**c**) tan δ_ε_ of PVDF/rGO/AgNWs−H, PVDF/rGO/AgNWs−L, and PVDF/rGO/AgNWs−B.

The electromagnetic parameters of the composite material are shown in Figure 7. As shown in Figure 7a, the real part (ε′) of PVDF/rGO/AgNWs−H (139.0–1.97) is higher than that of PVDF/rGO/AgNWs−L (47.1–0.573) and PVDF/rGO/AgNWs−B (57.8–12.6) because the high molding pressure crushed the AgNWs, and the heterojunction interface of PVDF/rGO/AgNWs−H increased, which enhanced the energy storage [30].

In the X-band, the imaginary part (ε″) reflects the electromagnetic wave dissipation characteristics of the material. As shown in Figure 7b, the ε″ of PVDF/rGO/AgNWs−B (224.7–118.9) was higher, followed by PVDF/rGO/AgNWs−L (146.5.0–2.15). The ε″ of PVDF/rGO/AgNWs−H (63.9–2.14) was smaller due to the excellent conductivity of the composite materials, the obtained conductive loss medium, and the interface polarization loss caused by the difference in conductivity and/or dielectric constant with the adjacent phases rGO, AgNWs, and PVDF [31]. Porous heterogeneous interfaces further increased the interfacial energy loss. In addition, the porous structure prolonged the loss path of electromagnetic waves and increased the dielectric loss capacity.

Comparing ε′ and ε″, the energy storage of PVDF/rGO/AgNWs−H was greater than the electromagnetic wave energy lost. Meanwhile, the energy loss of PVDF/rGO/AgNWs−L and PVDF/rGO/AgNWs−B was greater than the energy storage. The dielectric loss capacity of the PVDF/rGO/AgNWs−L and PVDF/rGO/AgNWs−B composite materials was much greater than their dielectric energy storage capacity. The PVDF/rGO/AgNW−L and PVDF/rGO/AgNW−B composite materials were the main loss mechanisms for converting electromagnetic energy into other forms of energy, such as heat. In addition, the ε″ of the composite materials exhibited significant fluctuations. As the frequency increases, the polarization process of the dielectric material may be unable to keep up with the changes in the external electric field promptly, resulting in an increased energy loss [32,33].

Figure 7c shows the dielectric loss (tan δε) of the composite material. The tan δε of PVDF/rGO/AgNWs−L (19.20–0.586) and PVDF/rGO/AgNWs−B (17.0–3.38) was relatively large, while that of PVDF/rGO/AgNWs−H (0.477–3.97) was relatively small. The absorption contribution can be significantly enhanced by various loss mechanisms via the increased internal porosity of low-pressure-molded and backfilled composite materials.

### 3.5. Mechanism Analysis

**Figure 8:**
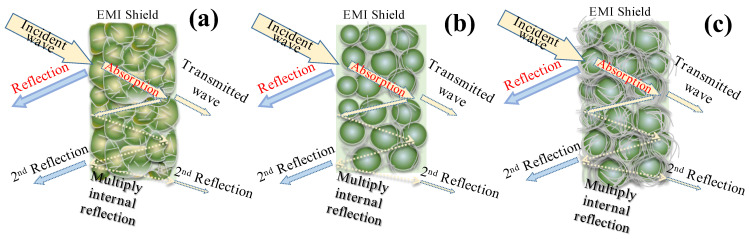
Schematic representation of the EMI shielding mechanisms for PVDF/rGO/AgNW composites with different structures: (**a**) compact and uniform; (**b**) porous structure; and (**c**) AgNW backfill porous structure.

The EMI shielding mechanisms for the PVDF/rGO/AgNW composites with different structures are depicted in Figure 8. As shown in Figure 8a, the polymer composite materials contained many heterogeneous interfaces, leading to the dissipation of incident EMW due to dielectric losses [34,35] (i.e., the polarization of multiple heterogeneous interfaces, dipole polarization, and conduction losses).

As shown in Figure 8b, increasing the porous structure of the material interface enhanced the multiple-reflection effect to enhance the internal reflection of the shielding material, i.e., the reflection on different surfaces or interfaces due to the non-uniformity within the prepared nanocomposite material [13]. The composite material of the conductive rGO sheet, AgNWs, and porous structure improved the impedance matching, created a microcurrent network, and achieved interface polarization [36,37]. As a result, electromagnetic waves underwent multiple internal reflections within the composite’s porous structure, converting the energy into thermal energy and weakening the waves’ propagation ability. Using porous materials can further enhance this effect [38] by increasing the interfaces in electromagnetic shielding composites.

The conductivity of PVDF/rGO/AgNWs−L was inferior to that of PVDF/rGO/AgNWs−H and PVDF/rGO/AgNWs−B (as depicted in Figure 5). Moreover, the low-pressure process impeded enhancements in conductivity and, consequently, the electromagnetic shielding effectiveness that conductivity affords. Nonetheless, the low-pressure process is essential for fabricating porous structures, which are pivotal in augmenting the electromagnetic shielding efficiency.

More importantly, the electromagnetic interference shielding and absorption contribution can be improved by introducing high-aspect-ratio AgNWs into the composite porous layer and optimizing the conducting network. As shown in Figure 8c, the composite backfilling of the porous structure with AgNWs increased the conductivity and enhanced its interaction with electromagnetic waves via high dielectric loss. The porous structure resulting from the backfilling of AgNWs increased the material’s interfacial area and multiple internal reflections. As a result, the conductivity loss and absorption contributions were significantly increased.

More importantly, when high-frequency electromagnetic waves are incident on a backfilled AgNW porous structure composite, a fraction of the energy is reflected by the highly conducting nanowires on the surface due to the impedance mismatch between the material and the free space. The porous structure of the surface helps to reduce impedance mismatch by allowing EM waves to enter the interior. When electromagnetic waves enter the material’s interior, backfilling AgNWs promote dipole polarization, resulting in dielectric loss at the material’s internal interface and the generation of eddy current loss transformed into thermal energy. In addition, the backfilling of AgNWs with nanowires of remarkable conductivity in a porous composite results in the formation of many microcavities in the region, which reduce the EM wave energy by accumulating and migrating space charges [29]. Microwaves are either reflected or absorbed within the material due to these synergistic effects, while only a few pass through it, increasing the absorption contribution.

The PVDF/rGO/AgNW composite is resistant to interference caused by electromagnetic waves. The PVDF/rGO/AgNW composite has several advantages compared with conventional metal electromagnetic shielding materials, such as good environmental tolerance, especially a strong loss capacity against electromagnetic waves, absorption contribution, and low secondary contamination. The PVDF/rGO/AgNW composite has potential applications in electromagnetic shielding to reduce electromagnetic wave contamination in precision instruments, medical devices, navigation devices, radio and television, and other devices [39,40,41].

## 4. Conclusions

We examined the electromagnetic interference shielding of PVDF/rGO/AgNW composites utilizing low-pressure fabrication techniques and filling strategies. Firstly, a PVDF/rGO/AgNW composite with a segregated structure was constructed using low-pressure molding. The silver nanowires maintained good dimensional stability during the preparation process. Afterward, the composite material was further processed using the vacuum-backfilling method. The AgNWs incorporated using the backfilling method facilitated the formation of a more comprehensive conducting network and significantly increased the conductivity to 1.07 S/m. In addition, the composite’s SE_T_ was increased from 24.88 dB to 30.67 dB after backfilling with 1 wt% silver nanowires, representing an improvement of 23.3%. Introducing silver nanowires also significantly promoted multiple internal reflections and extended the conduction loss process, thus enhancing the absorption capacity of electromagnetic waves. The absorption contribution (SE_A_/SE_T_) increased from 84.2% to 92.8%, indicating a notable enhancement in the absorption-dominated electronic shielding efficiency of the composite material. These findings favor the fabrication of PVDF/rGO/AgNW composites for advanced EMI shielding applications with optimized performance. The PVDF/rGO/AgNW composite has potential applications in electromagnetic shielding to reduce electromagnetic wave contamination in precision instruments, medical devices, navigation devices, radio and television, and other devices.

## Data Availability

The data presented in this study are available on request from the corresponding author.
